# Application of an instructive hydrogel accelerates re-epithelialization of xenografted human skin wounds

**DOI:** 10.1038/s41598-022-18204-w

**Published:** 2022-08-20

**Authors:** Holly D. Sparks, Serena Mandla, Katrina Vizely, Nicole Rosin, Milica Radisic, Jeff Biernaskie

**Affiliations:** 1 Department of Comparative Biology and Experimental Medicine, Faculty of Veterinary Medicine, University of Calgary, Calgary, AB T2N 4N1 Canada; 2 Toronto General Research Institute, University of Toronto, Toronto, Canada; 3 Institute of Biomaterials and Biomedical Engineering, University of Toronto, Toronto, Canada; 4 Department of Chemical Engineering and Applied Chemistry, University of Toronto, Toronto, Canada; 5 Alberta Children’s Hospital Research Institute, Calgary, AB Canada; 6 Hotchkiss Brain Institute, Calgary, AB Canada

**Keywords:** Medical research, Engineering

## Abstract

Poor quality (eg. excessive scarring) or delayed closure of skin wounds can have profound physical and pyschosocial effects on patients as well as pose an enormous economic burden on the healthcare system. An effective means of improving both the rate and quality of wound healing is needed for all patients suffering from skin injury. Despite wound care being a multi-billion-dollar industry, effective treatments aimed at rapidly restoring the skin barrier function or mitigating the severity of fibrotic scar remain elusive. Previously, a hydrogel conjugated angiopoietin-1 derived peptide (QHREDGS; Q-peptide) was shown to increase keratinocyte migration and improve wound healing in diabetic mice. Here, we evaluated the effect of this Q-Peptide Hydrogel on human skin wound healing using a mouse xenograft model. First, we confirmed that the Q-Peptide Hydrogel promoted the migration of adult human keratinocytes and modulated their cytokine profile in vitro. Next, utilizing our human to mouse split-thickness skin xenograft model, we found improved healing of wounded human epidermis following Q-Peptide Hydrogel treatment. Importantly, Q-Peptide Hydrogel treatment enhanced this wound re-epithelialization via increased keratinocyte migration and survival, rather than a sustained increase in proliferation. Overall, these data provide strong evidence that topical application of QHREDGS peptide-modified hydrogels results in accelerated wound closure that may lead to improved outcomes for patients.

## Introduction

Skin is the largest organ in the body and is responsible for a range of vital functions such as thermoregulation, tactile sensation, and synthesis of vitamins and metabolites. Perhaps most importantly, it also acts as a physical barrier; protecting the body against bacterial invasion and preventing fluid loss^1^. Wounds, or disruption of this physical barrier, have a wide variety of causes including surgical interventions, acute traumatic injury, and underlying conditions such as diabetes or advanced age predisposing the patient to tissue loss secondary to otherwise normal extrinsic forces such as pressure or shear^2^. Irrespective of cause, the hallmark of clinical wound healing or “closure” is complete restoration of the epithelium, a process carried out by a combination of wound contraction and regeneration of the epithelium (re-epithelialization). In humans, up to 80% of wound closure is attributed to re-epithelialization^3^. During re-epithelialization, keratinocytes at the wound margin undergo three overlapping events: migration, proliferation, and differentiation. Of these events, the most consistent limiting step is that of keratinocyte migration^4^. Thus, one effective method of improving the rate of wound closure through re-epithelialization in humans would be through the modulation of keratinocyte migration.

Indeed, poor wound healing remains a clinical challenge for both patients and health professionals globally; and cost-effective, safe, and reliable methods of improving wound healing remain elusive. In the United States alone, more than 6 million people are affected by chronic wounds every year leading to an estimated $20–25 billion spent in healthcare^5^. One promising approach toward the development of materials capable of improving human wound healing involves harnessing the body’s natural growth factors to promote tissue repair and regeneration. However, this area of research is often hindered by molecule stability during delivery, practicality of application, and high costs. More recently, sophisticated polymer and material chemistry has allowed for the functionalization of various peptides and proteins to develop “smart biomaterials”, yielding products that can interact with the local cellular environment to provide instructional cues, but with higher stability during delivery, increased specificity and localized effects, and lower overall cost for production^6^. Of particular interest is the QHREDGS peptide (“Q-Peptide”), which is an angiopoietin-1 mimetic peptide found in the fibrinogen domain of angiopoietin-1 that is largely conserved amongst species. In some cell types such as endothelial cells, angiopoietin-1 functions through a tyrosine kinase receptor, Tie2^7^. However, angiopoietin-1 can also promote the adhesion and survival of cells such as fibroblasts, which lack the Tie2 receptor, leading researchers to implicate integrin binding in the function of angiopoietin-1^8^. Since angiopoietin-1 lacks an RGD site typically required in integrin binding, Dallabrida et al. examined the sequence of angiopoietin-1 for regions resembling integrin motif. The QHREDGS sequence within angiopoietin-1 was eventually identified due to its similarity to KRLDGS, a fibrinogen integrin motif, and REDV, a fibronectin motif^8^. It was later determined that the QHREDGS sequence interacts with a number of different integrins, but primarily that of the β_1_ type^9–11^. Given that β_1_-integrins play a crucial role in keratinocyte migration and wound healing, we were motivated to study the effect of the Q-Peptide on accelerating and improving healing of human skin^12^. In the current study, this Q-Peptide was conjugated to chitosan and mixed with collagen, to create a hydrogel capable of the in vivo delivery of the Q-Peptide in a moist wound healing environment^13^, which in itself is known to promote improved re-epithelialization^14^.

Recently, Xiao et al. demonstrated the efficacy of this Q-Peptide collagen-chitosan hydrogel in promoting neonatal human keratinocyte (HEK) migration in vitro, as well as protecting against harmful reactive oxygen species in culture, which are frequently generated in chronic wounds. When used in an excisional wound in a diabetic mouse model, the peptide hydrogel also significantly accelerated re-epithelialization and closure of the wound, while increasing granulation tissue formation^15^. In addition to the diabetic mouse wound healing model, we demonstrated improved wound healing in equine lower limb wounds with the treatment of the Q-Peptide Hydrogel. Equine skin treated with the Q-Peptide Hydrogel resulted in a higher rate of healing and more compliant tissue^16^. These two bodies of work provided valuable insight into the safety and utility of the Q-Peptide Hydrogel in both small and large animal models of impaired wound healing, however questions remain as to the efficacy of the Q-Peptide Hydrogel on wound healing in human skin.

Herein, we hypothesized that the Q-Peptide Hydrogel would support the survival and migration of adult human keratinocytes and ultimately speed re-epithelialization to improve healing of wounds in human skin. To investigate this, we utilized adult human keratinocytes to compare the effects of the Q-Peptide Hydrogel to both a peptide-free and scrambled peptide hydrogel on cell migration, proliferation, and cytokine production in vitro. Next, we utilized a human to mouse split thickness xenograft model to determine the effect of the Q-Peptide Hydrogel on closure of wounds in adult human epidermis. Last, we explored the quality of healing at three timepoints across the course of wound healing.

## Results

### Q-Peptide Hydrogel promotes accelerated closure through increased keratinocyte migration and altered secretory profile

**Figure 1:**
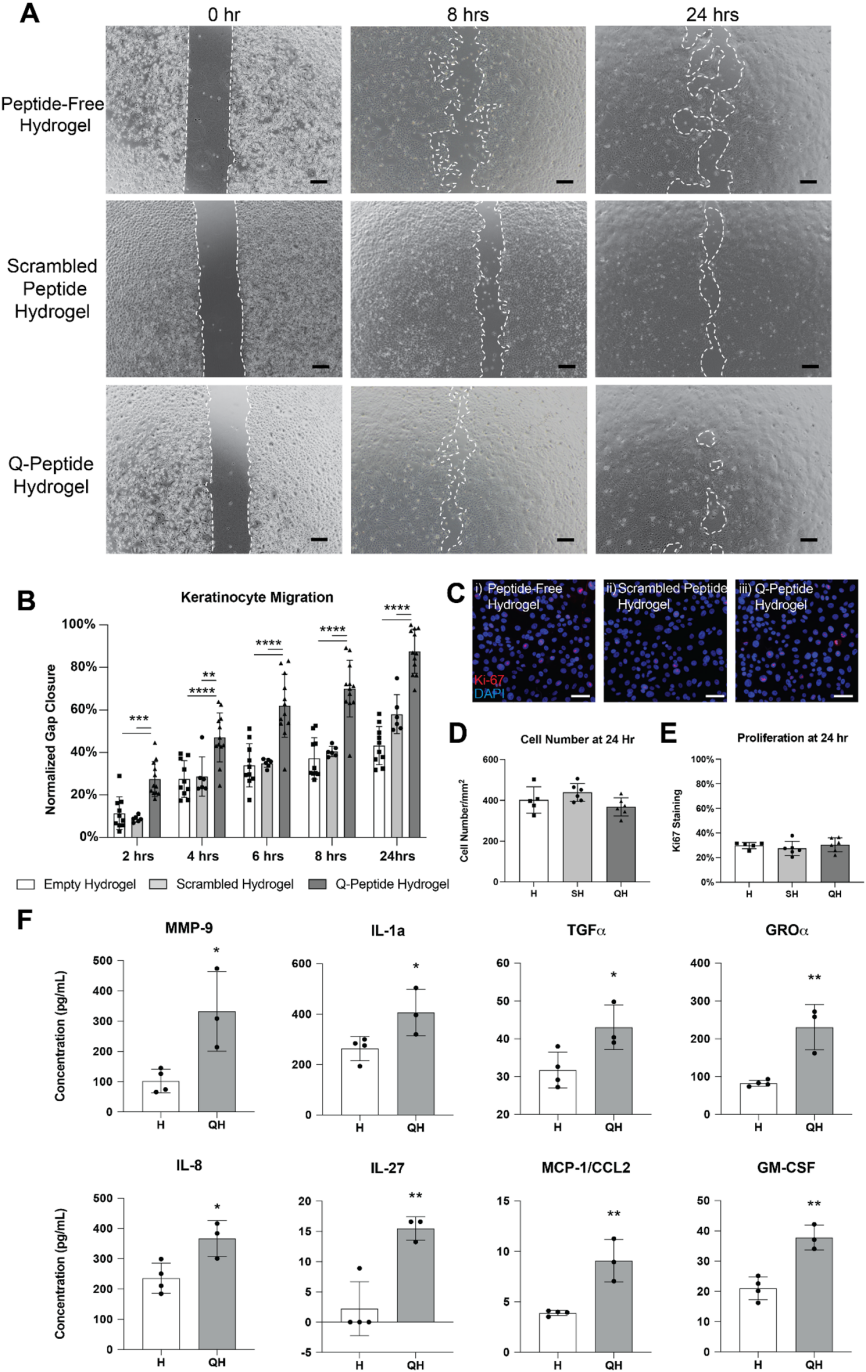
Accelerated normal adult human keratinocyte (HEKa) migration and altered cytokine profile are observed on QHREDGS peptide conjugated collagen-chitosan film (Q-Peptide Hydrogel). (**A**) Representative examples of wound simulation assay using HEKa cells adhered to hydrogel film without conjugated peptide, with scrambled Q-Peptide, and with QHREDGS peptide after 24 h (scale bars: 200 μm). (**B**) HEKa migration (relative to initial gap area) in the presence of Q-Peptide Hydrogel (dark grey; n = 12) was accelerated in comparison to the collagen-chitosan film without peptide conjugation (Peptide-free hydrogel; white; n = 11), and the scrambled peptide Hydrogel (light grey; n = 6). (**C**) Representative images of HEKa cells stained with Ki-67 (red) and counterstained with DAPI (blue) after 24 h (scale bars = 100 μm). (**D**) Quantification of cell number as indicated by DAPI staining demonstrates no difference in cell density at 24 h. (**E**) Quantification of % positive Ki-67 staining revealed no significant difference between cells cultured on the Q-Peptide Hydrogel (QH) and the peptide-free hydrogel. (**F**) Human cytokine array on media collected after migration assay conducted on Peptide-free hydrogel and Q-Peptide Hydrogel. Concentration (pg/mL) expressed after subtraction of baseline media. n = 3. H = Peptide-Free Hydrogel, SH = Scrambled Peptide Hydrogel, QH = Q-Peptide Hydrogel. Data are presented as mean ± SD. **p < 0.01.

Using adult human epidermal keratinocytes, we observed a faster gap closure on Q-Peptide Hydrogel film compared to the Peptide-Free Hydrogel and the Scrambled (DGQESHR) Peptide Hydrogel indicating a significantly greater keratinocyte migration as early as 2 h (Fig. 1A). Upon study completion (24 h), keratinocytes cultured on Q-Peptide Hydrogel film were observed to close 88% (± 10%) of the gap, whereas those cultured without the Q-Peptide or on the Scrambled Peptide film displayed only a 43% (± 9%) and a 54% (± 4%) closure, respectively (Fig. 1B). No significant cell death was observed throughout the 24 h as confirmed by LDH assay (Supplementary Figure 1). Ki-67, a marker of proliferating cells, demonstrated no significant difference between the proliferation of migrating keratinocytes on the Q-Peptide Hydrogel films, the Peptide-free films and the Scrambled Peptide film after 24 h, suggesting that sustained proliferation did not accompany accelerated wound closure (Fig. 1C,D,E, Supplementary Figure 2). We further validated that the Q-Peptide Hydrogel does not directly promote cell proliferation by culturing human dermal fibroblasts, another important skin cell type, with and without the Q-Peptide Hydrogel. No significant difference was observed after 24 h in Ki-67 staining between the two conditions (Supplementary Table 1).

To explore the mechanism through which the Q-Peptide Hydrogel affects keratinocyte function and how they might influence other cells within the wound to improve wound healing,focused on identifying key cytokines at play (Fig. 1F). In light of our keratinocyte migration assay, where no functional effect of the scrambled peptide was observed and numerous previous studies demonstrating no efficacy of the scrambled peptide^11,13,17–20^, we elected to exclude this control group from all experiments going forward. Remarkably, keratinocytes responding to gap creation in the presence of the Q-Peptide Hydrogel produced significantly greater concentrations of several key cytokines known to be critically involved in wound healing by enabling matrix remodelling (MMP-9), activating keratinocytes and promoting their migration ( IL-1a, TGFα, CxCl1 (GROa), IL-8, and IL-27), contributing to re-epithelialization by supressing terminal differentiation of keratinocytes (IL-27) and by contributing to the acute inflammatory phase of wound healing that initiates processes such as revascularization (MCP-1, GM-CSF and IL-8) (Fig. 1F).

Further, the solvent casted films (both peptide-free and Q-Peptide) were found to be hydrophilic as evidenced by a contact angle of < 90°, which decreased over time of water-droplet exposure (Supplementary Figure 4A). Further, both hydrogels were demonstrated to be water swollen after 24 h, increasing to 4–6 times their original weight (Supplementary Figure 4B). This confirms that the hydrogel surfaces are suitable for cellular attachment and motility, and generally improve wound healing by providing a moist environment (Supplementary Figure 4B).

### A human to mouse split-thickness skin xenograft recapitulates human epidermal healing

**Figure 2:**
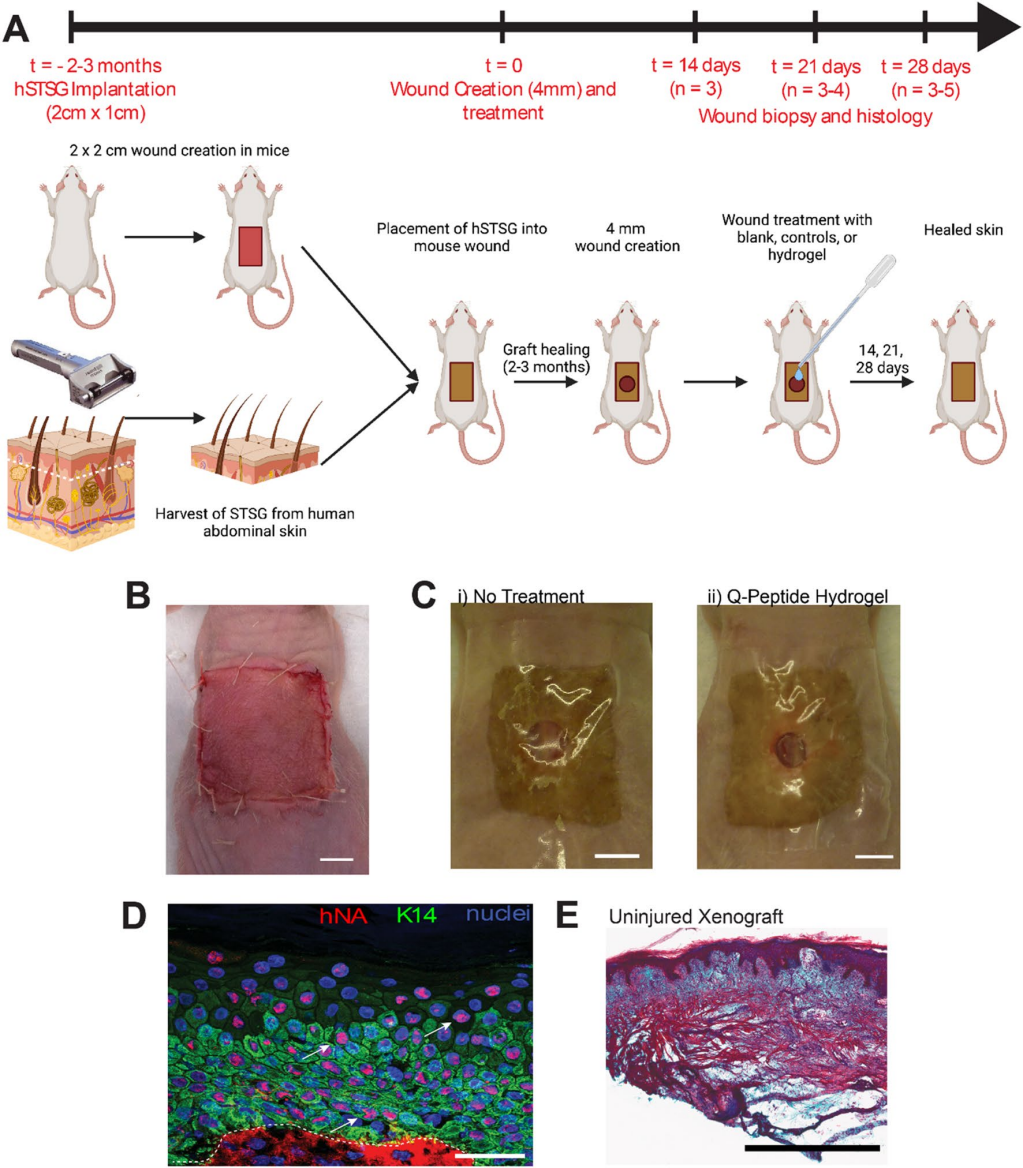
Wounds created in adult human split thickness skin xenografts display evidence of re-epithelialization with human epidermis. (**A**) Split thickness xenograft model. (**B**) Split thickness xenografts were harvested from donor human skin and grafted onto 2 × 2 cm full thickness defects created on the backs of nude (Nu/Nu) mice (scale bar = 0.5 cm). (**C**) After 3 months, accepted hSTSG were then wounded with a 4 mm biopsy punch and treatments immediately applied then maintained by covering with a semi-permeable adherent dressing (Tegaderm) throughout the duration of wound healing (scale bar = 0.5 cm). (**D**) Immunohistochemistry of healed wounds confirmed presence of human epidermis through double staining for keratin 14 (K14) to generally identify keratinocytes and human nuclear antigen (hNA, arrows) to specifically identify human keratinocytes in the center of a healed wound. Dashed line indicates basal epithelial layer (scale bar = 50 µm). (**E**) Masson’s Trichrome stained uninjured xenograft demonstrating the fibrotic dermis that is typically associated with this model. Scale bar = 1 mm. *Created with Biorender.com*.

We set out to explore the efficacy of the Q-Peptide Hydrogel specifically on epidermal migration of human cells in an in vivo environment. To test this, we utilized a human to mouse split thickness skin xenograft where the epidermis and a thin layer of the dermis are harvested from donor human skin and grafted onto a full thickness defect created on the back of an immunocompromised (Nu/Nu) mouse^21,22^ (Fig. 2A–C). To account for human donor variability, experiments were conducted such that each replicate included herein (n = 3–5 per analysis) represents a different human skin donor and these same human donors were included as biological replicates across each treatment group, by wounding (4 mm diameter) the center of the healed graft, 2–3 months after grafting. Human nuclear antigen staining confirmed the presence of human epidermis in this model, ensuring that wound closure in the xenograft was the result of human epithelial cell coverage (Fig. 2D) and not due to recruitment of mouse cells. As previously described, at the time of wounding, the dermis underlying the human epidermal graft was found to be comprised exclusively of mouse cells characterized by disorganized fibrotic tissue devoid of hair follicles and other adnexa normally present in the dermis (Fig. 2E).

### Q-Peptide Hydrogel accelerates wound closure without increasing wound contraction

**Figure 3:**
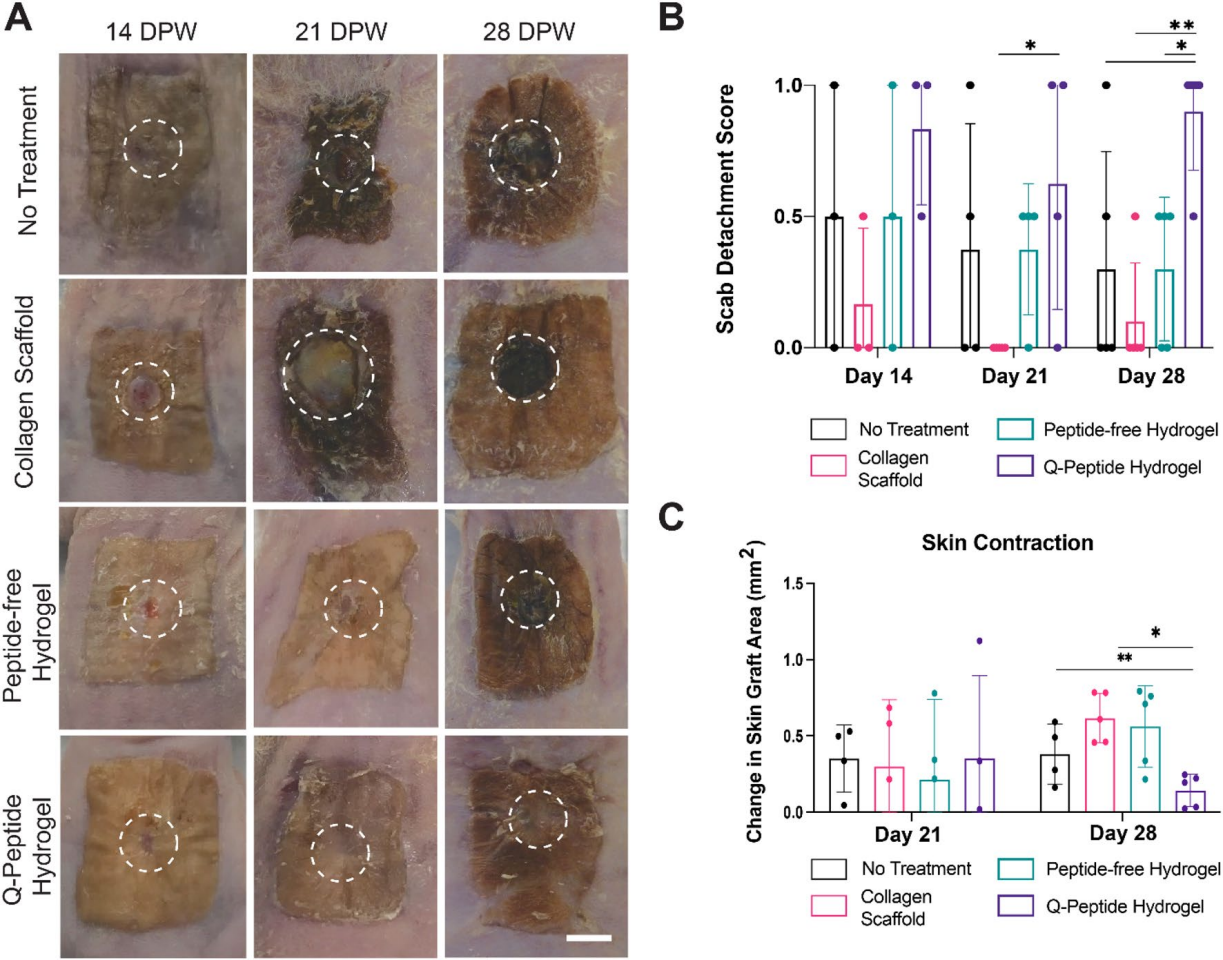
Treatment with QHREDGS peptide conjugated hydrogel speeds wound closure without increasing the amount of wound contraction. (**A**) Gross photographs of wounds at 14, 21, and 28 days post wounding (DPW). Representative images across timepoints are from three separate biological replicates (scale bar = 500 mm). (**B**) Wound appearance was scored using a scab detachment score where open wounds, profound scab = 0, closed wound with scab contraction = 0.5, and closed wound, no scab present = 1. Q-Peptide Hydrogel treated wounds showed significantly improved wound score on day 28. (**C**) Measurement of the change in overall graft area after 21 and 28 days indicated significantly less healing occurred through wound contraction of Q-Peptide Hydrogel treated wounds compared with peptide-free hydrogel and collagen scaffold controls. E = No treatment, C = collagen scaffold, H = Peptide-free hydrogel, QH = Q-Peptide Hydrogel Data are presented as mean ± SD. *p < 0.05, **p < 0.01. N = 3–5.

Gross morphological images of the treated wounds taken at the time of euthanasia indicated a more complete wound closure in the Q-Peptide Hydrogel group compared with controls (Fig. 3A). By day 28, the wounded area can still be seen in the no treatment and collagen scaffold controls, while the Q-Peptide Hydrogel nearly eliminated the appearance of the wound, suggesting a more advanced stage of wound closure. Scoring of scab resolution indicated that the wounds treated with the Q-Peptide Hydrogel scored significantly higher than all other treatment groups, with all but one wound (1/5) reaching full closure with no scab present by day 28 (Fig. 3B). No significant differences in wound contraction were observed among the treatment groups at Day 21, as assessed by the change in total graft area (Fig. 3C). Importantly, at Day 28 significantly less wound contraction was observed for Q-Peptide Hydrogel treated wounds when compared with Peptide-free hydrogel and the collagen scaffold (Fig. 3C).

**Figure 4:**
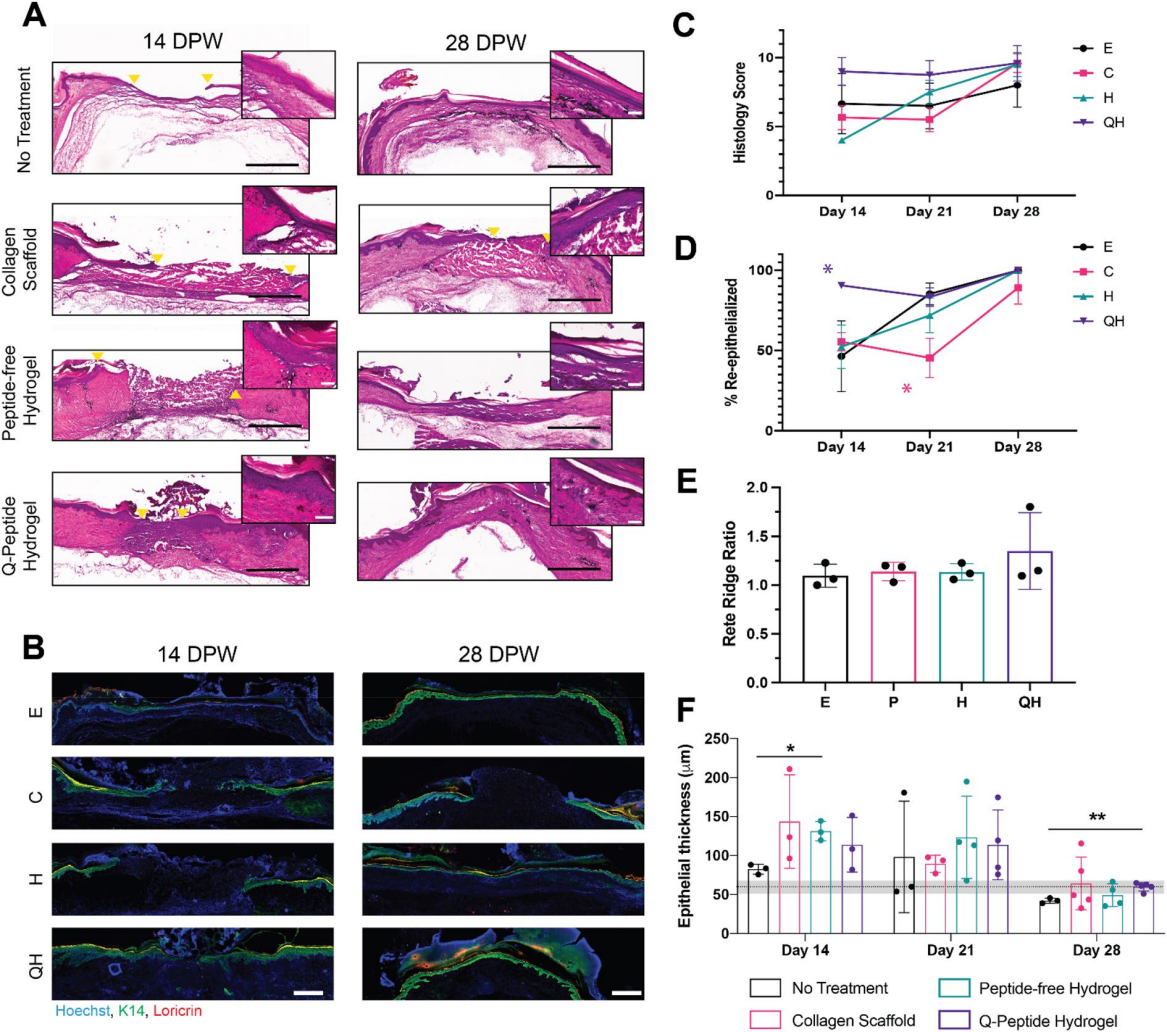
Histology of healing wounds reveals accelerated wound re-epithelialization with Q-Peptide Hydrogel treatment. (**A**) Representative Hemotoxylin and Eosin stained cryosections from the middle of wounds at 14, and 28 days post wounding (DPW). Yellow arrow heads indicate measurements used for wound gap. SB = 1 mm. Inset SB = 0.1 mm. (**B**) Immunostained tissue sections on day 14 and day 28 for K14 (Green), Loricin (Red) and Hoechst (Blue). SB = 500 μm. (**C**) Qualitative histology scoring (1–12, with higher scores indicating greater granulation tissue filling, re-epithelialization, and reduction of cellular infiltration) shows a trend (*p* = 0.09) toward faster and more complete wound healing following treatment with Q-Peptide Hydrogel. (**D**) Significantly faster re-epithelialization of wounds treated with Q-Peptide Hydrogel was observed at earlier timepoints (days 14 and 21, *p* < 0.05). (**E**) Measured rete ridge ratio at D28 for no treatment, collagen scaffold, Peptide-free hydrogel, and Q-Peptide Hydrogel. (**F**) Measurement of epithelial thickness in healing wounds revealed thickening of neoepidermis in all groups at early timepoints (day 14 and 21) compared with uninjured human skin (dashed line +/− SD, area shaded in grey). Treatment with Q-Peptide Hydrogel promoted more complete return to pre-injury epidermal thickness by day 28 compared with no treatment control. *p < 0.05, *p < 0.05 (collagen scaffold significantly less than other treatments) **p < 0.005, E = No treatment, C = collagen scaffold, H = Peptide-free hydrogel, QH = Q-Peptide Hydrogel, N = 3–5.

Tissue sections from the middle of the wound were stained with H&E (Fig. 4A, Figure S5A). As we utilized relatively thick (35 μm) cryosections in this study so as to optimize our immunohistochemistry applications, tissue architecture and intensity of H&E staining is notably poorer than achieved with paraffin-embedded sections. To overcome this and confirm the accuracy of our measurements of wound healing, we stained neighboring sections with K14 and loricrin to label mature epidermis^23^ (Fig. 4B, Figure S5B). Based on a previously published scoring of H&E sections, ranging from 1 (no healing) to 12 (complete healing), to encompass features of wound healing such as the presence and organization of granulation tissue, persistence of inflammation, neovascularization, and re-epithelialization^24^, we observed a trend (*p* < 0.09) for improved rate and quality of wound healing following treatment with Q-Peptide Hydrogel, specifically at an early time point of day 14 (Fig. 4C). Additionally, by day 14, wounds treated with the Q-Peptide Hydrogel displayed significantly greater re-epithelialization of the wound (mean 90.6 +/− 1.0%) compared to no treatment (46.4 +/– 31.2%), collagen scaffold (55.54 +/− 9.6%), and peptide-free hydrogel (52.36 +/− 23.41%) (Fig. 4D). This was also evident in the K14 staining which extended almost the entire wound surface following Q-peptide treatment (Fig. 4B). Conversely, by day 21, the collagen scaffold significantly reduced re-epithelialization compared with the no treatment control and Q-Peptide hydrogel. At day 28, only 60% of wounds treated with the collagen scaffold (3/5) exhibited complete re-epithelialization compared with 100% of wounds in all other treatment groups (Fig. 4D).

No significant differences in the rete ridge coverage was observed amongst the groups, although Q-Peptide Hydrogel treated wounds exhibited a slightly greater average number compared to the other treatment groups (Fig. 4E, Supplementary Figure 7). Next, when compared to uninjured adult human epidermis (59.9 +/− 8.7 µm^25^), an observable increase in thickness of the neoepidermis is present across all groups during early wound healing (day 14–21) (Fig. 4F). At day 28, a significant thinning was identified in the no treatment control compared with Q-Peptide Hydrogel treated wounds and a wide range of thicknesses were recorded for the collagen scaffold treatment. Conversely, wounds treated with Q-Peptide Hydrogel most closely approximated that of uninjured adult human epidermis (Fig. 4F). However, it should be noted that the collagen scaffold and the Peptide-free Hydrogel treated wounds had epidermal thicknesses similar to normal human epidermis by day 28, suggesting the addition of a hydrogel or collagen scaffold may help to promote healthy epidermal thickness maturation.

### Q-Peptide application does not lead to sustained proliferation of basal epithelial cells

**Figure 5:**
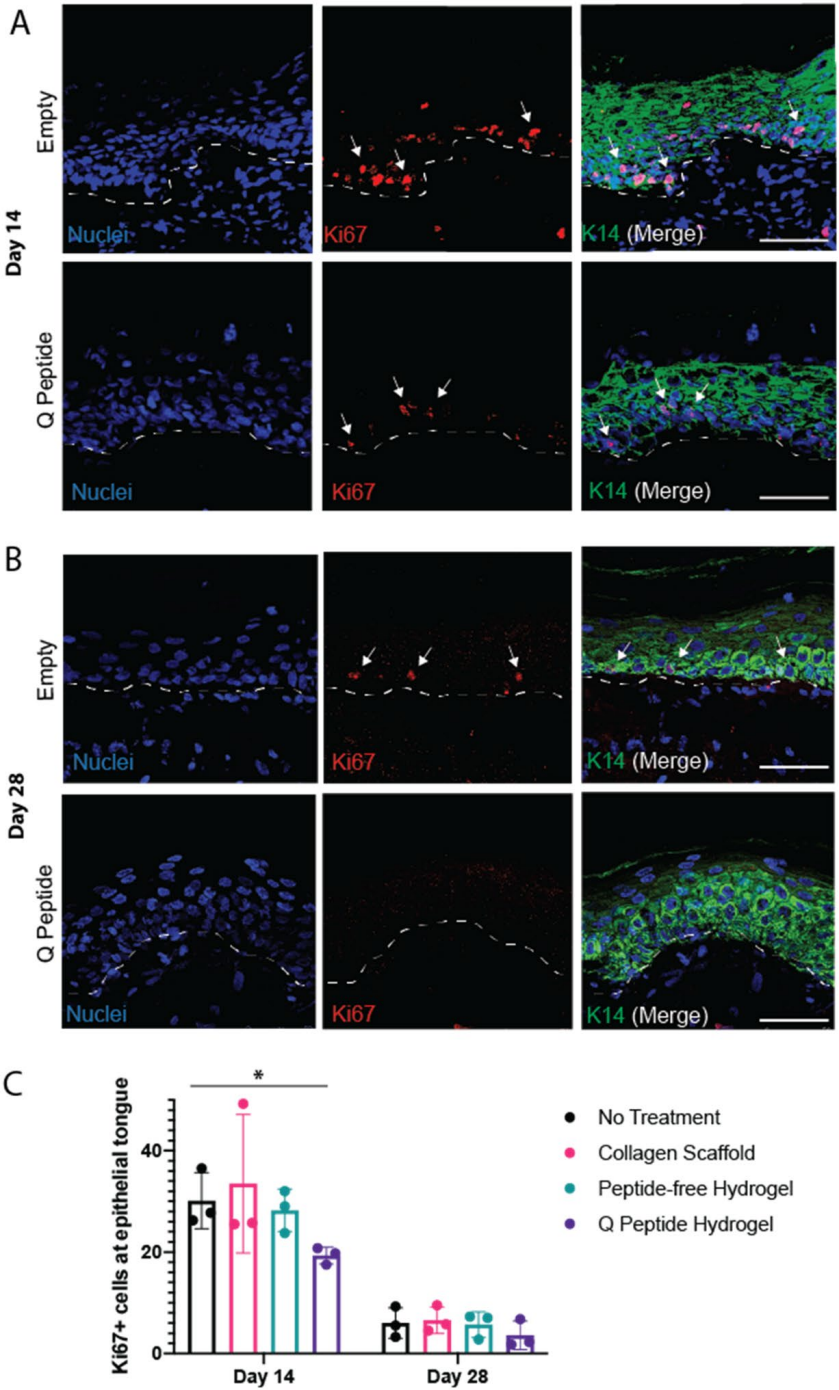
Treatment with Q-Peptide Hydrogel leads to faster resolution of proliferating keratinocytes at the margin of the wound. Representative sections of immunohistochemical analysis for Ki67 at (**A**) d14 and (**B**) d28. Dashed line indicates basal epithelial layer. Arrows show Ki67 positive nuclei. (scale bar = 100 µm). (**C**) Quantification of total number of Ki67+ cells in one high powered field of view at the epithelial edge (averaged from 4 separate tissue sections spanning the center of the wound) revealed significantly fewer proliferating basal epithelial cells on Day 14 following treatment with Q-Peptide Hydrogel compared with no treatment control. Data are presented as mean ± SD. *p < 0.05, N = 3.

The mechanism underlying the increased rate and quality of re-epithelialization in Q-Peptide Hydrogel treated wounds was explored by evaluating proliferation of basal epithelial cells in the center of the wound. Significantly fewer proliferative cells (Ki67+) were demonstrated in Q-Peptide Hydrogel treated wounds compared with no treatment control at Day 14 (Fig. 5A,C, Supplementary Figure 6). Importantly, the expected overall increase in proliferation was observed across all groups at Day 14 consistent with early wound healing (Fig. 5C). By day 28, proliferation decreased toward baseline across all groups indicating no evidence of a sustained hyperproliferative response to wound healing in any treatment groups (Fig. 5C).

### Q-Peptide Hydrogel stimulates neovascularization in healing wounds

**Figure 6:**
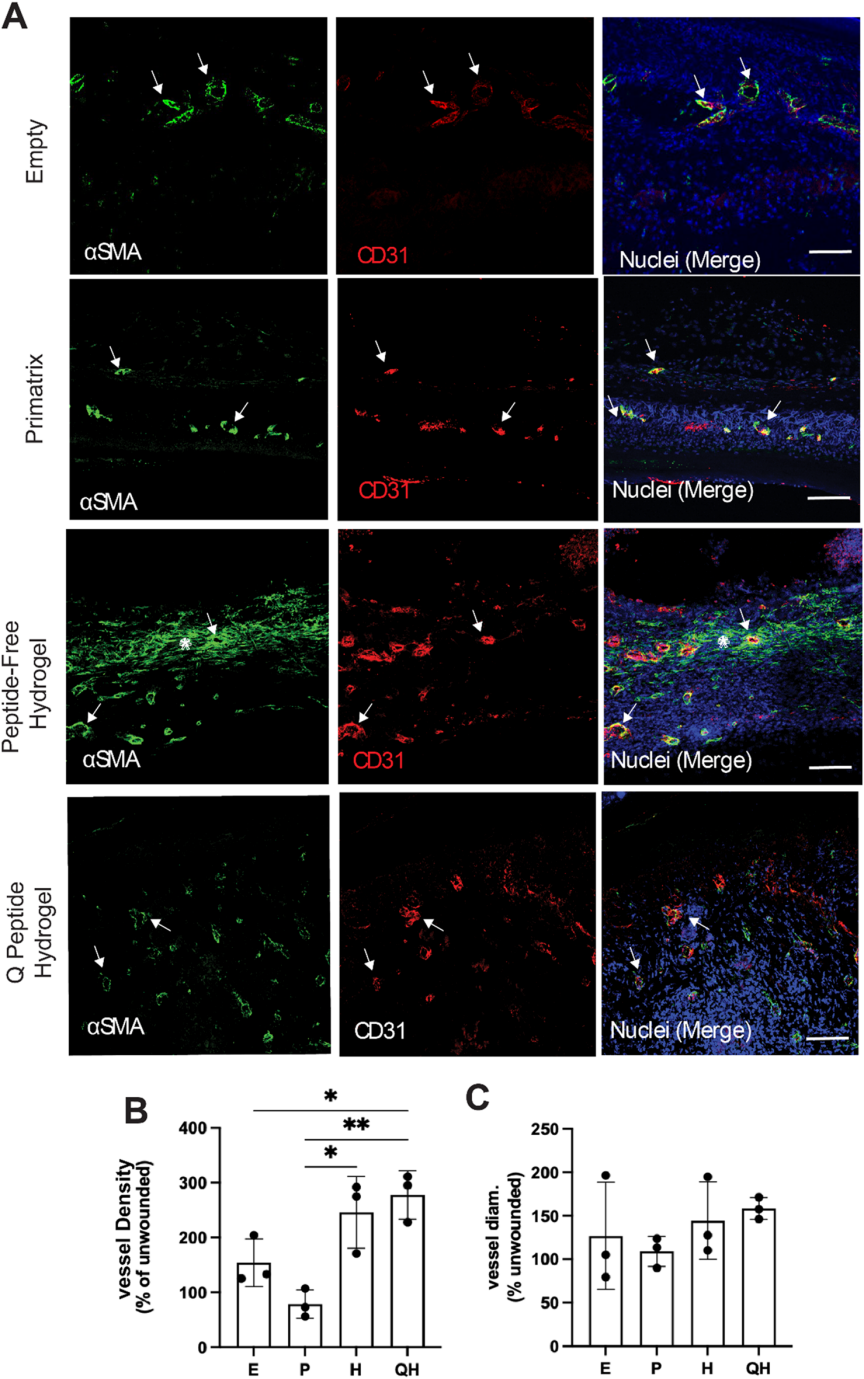
Treatment with Q Peptide Hydrogel increases neovascularization of healing wounds at day 28. (**A**) Immunofluorescence staining of the central region of day 28 wounds for α-smooth muscle actin (αSMA) and CD31. Arrow, double-positive stained vessels. Asterix, single positive (αSMA or CD31) staining unassociated with vessels. Scale bar 100 µm. (**B**) Quantification of blood vessel density normalized to the non-wounded area shows increased neovascularization in Q-Peptide treated wounds relative to no treatment and collagen scaffold controls. (**C**) Quantification of blood vessel diameter shows no significant difference across treatment groups. Data are presented as mean ± SD. *p < 0.05. E = No treatment, C = Collagen Scaffold, H = Peptide free Hydrogel, QH = Q-Peptide Hydrogel (n = 3).

Neovascularization, or the formation of new blood vessels, is a prerequisite of wound healing and abnormal angiogenesis is observed in nearly all chronic wounds^26^. Angiopoietin-1, one of the endogenous proteins mimicked by the Q-Peptide, has repeatedly shown a role in increasing neovascularization during wound healing in mice^27,28^. With this in mind, we aimed to explore the effect of the Q-Peptide Hydrogel in the human to mouse split thickness xenograft wound model. The total number of blood vessels present within wounds treated with Q-Peptide Hydrogel was significantly higher than in the no treatment control and collagen scaffold (*p* < 0.05). A significant increase was also observed in both the Peptide-free and Q-Peptide hydrogels relative to the collagen scaffold alone. However, a lack of significant difference between the Q-Peptide and Peptide-free Hydrogel was observed, suggesting a portion of this effect on neovascularization is the result of the hydrogel itself and not specifically of the peptide (Fig. 6B). No significant differences were observed in the diameter of vessels across groups (Fig. 6C). The presence of α-SMA positive cells outside of blood vessel structure could be indicative of myofibroblasts, which showed the trend of being less abundant in the Q-peptide hydrogel, compared to the Peptide-free hydrogel (Supplementary Figure 8).

## Discussion

We have previously demonstrated that the use of the Q-Peptide Hydrogel led to improved wound healing outcomes and was well-tolerated in two widely-accepted animal models of aberrant wound healing, a diabetic mouse wound model^15^, and a lower limb equine model^16^, as well as directly on key immune cells involved in wound healing^17^. Here, we sought to explore the impact of this conjugated peptide on normal adult human keratinocytes and human epidermis. Previous biomaterial characterization demonstrated long-term hydrogel stability and minimal degradation under hydrolytic conditions, collagenase, and lysozyme^17^, a storage and loss modulus appropriate for soft tissues^29^, and similar surface features, imaged with scanning electron microscopy, between the Q-Peptide Hydrogel and the Peptide-free hydrogel^29^. In the current work we also further characterized hydrophilicity of the peptide-free and Q-Peptide materials, as well as the swelling equilibrium. Taken together, biomaterial characterization demonstrated that Q-Peptide conjugation does not significantly change biomaterial properties, and that it is suitable for wound healing applications.

Prior to treatment of human split-thickness skin grafted on the back of mice, we sought to ask whether enhanced gap closure of adult human keratinocytes was indeed mediated through the promotion of keratinocyte migration rather than sustained proliferation. Interestingly, culture atop the Q-Peptide conjugate hydrogel promoted the production of pro-wound healing cytokines from keratinocytes which likely contributed to this enhanced wound closure seen in vivo. Specifically, MMP-9, an enzyme known to transiently increase with wound healing, has previously been demonstrated as the key protease responsible for degradation of the matrix in chronic wounds^30,31^. Likewise, IL-1a, a known activator of tissue plasminogen activator (tPA), enhances pericellular proteolysis of keratinocytes along the advancing epithelial front, further aiding the migration of keratinocytes to close the wound^32^. IL-1a is additionally one of the most common activators of keratinocytes. Activated keratinocytes not only show enhanced migratory capabilities, but also produce other growth factors and cytokines essential to wound healing and display an increased level of cell surface receptors^33^. This feature certainly highlights the interplay between direct effects of the Q-Peptide Hydrogel and subsequent effects secondary to modulation of the initial signalling milieu. Additional factors that are significantly increased after migration on the Q-Peptide Hydrogel include TGFα, CxCl1 (GROa), IL-8, and IL-27 which are known promoters of keratinocyte migration^34–36^. TGFα, in particular, is so consistent in its ability to enhance keratinocyte migration that it is regularly used as a media additive in an experimental model to induce HEKa migration^33,37^. IL-27, also increased in keratinocytes when cultured on the Q-Peptide Hydrogel, has an essential role as a suppressor of terminal differentiation of keratinocytes during re-epithelialization^38^. In addition to known positive effects on keratinocytes, some cytokines found to be increased in keratinocytes cultured on the Q-Peptide Hydrogel also have known roles during dermal wound healing and inflammation. MCP-1, for instance, is a vital chemokine during the inflammatory phase of wound healing, that attracts monocytes/macrophages to the site of injury^35^. Granulocyte–macrophage stimulating factor (GM-CSF) also plays an important role as an immunomodulator; activating macrophages as well as mediating the maturation of langerhans and dendritic cells^39^. This chemokine, too, has demonstrated efficacy in promoting a regenerative epidermal phenotype when injected into skin^39^. Certainly, the altered cytokine profile observed here may represent a multi-faceted response of adult human keratinocytes to the Q-Peptide Hydrogel where the conjugated peptide induces an improved wound healing response phenotype aided by an increase in cytokines known to have positive effects on keratinocyte migration and wound healing.

After confirming direct effects on adult human keratinocytes, we next modified our human to mouse split thickness xenograft model^21,22^ to provide an in vivo assay of the effects of the Q-Peptide Hydrogel on human epidermis. Unique to standard rodent models of wound healing, which typically close wounds primarily through contraction as a result of their loose skin and the *panniculus carnosus*^40^, this xenograft model was observed to heal primarily through re-epithelialization and to a lesser degree by wound contraction, as is typically observed in human wound healing*.* Importantly, obtaining sufficient amount of skin from healthy adults undergoing abdominolasty allowed us to graft a minimum of 5 nude mice from the same adult, which was important for assessing different treatment options for the cutaneous wounds of the same human biological donor as well as the investigation of multiple biological replicates (human donors) within each treatment group, across all timepoints with a specific focus on re-epithelialization.

The hydrogels were applied to the wound in such a way to fill it completely from edge to edge, which is a critical step for the desired outcome of improved wound healing, thus the thickness of the swollen hydrogel is identical to the depth of the wound. Otherwise, the presence of gaps between the hydrogel and the wound edge could inhibit epithelial cell migration.

Fibrosis is a common event after split thickness grafting even with autologous grafts^41^**.** As a result, the fibrotic tissue has a potential for a significantly decreased microcirculation and thus nutrient and oxygen supply to the wound, which is one of the limitations of the current model and may negatively affect the outcomes of the wound healing process. While not representative of human dermis, this model may have the additional benefit of providing a surrogate means of studying wound healing in the presence of established dermal fibrosis such as that experienced in human patients living with split-thickness skin grafts^21^. For these patients, re-injury of the graft site is common and delayed or pathologic wound healing is commonly observed^42^. Indeed, a significant increase in neovascularization of this fibrotic dermis observed in both hydrogel treated groups may become a promising strategy for the treatment of chronic or fibrotic wounds which commonly exhibit deficiencies in vascular supply^43^.

Using this model, we confirmed that the Q-Peptide Hydrogel accelerates wound closure compared with controls. Surprisingly, our work identified slower re-epithelialization in wounds treated with the FDA-approved collagen scaffold control. This particular dermal repair scaffold is derived from fetal bovine dermis and is rich in type III collagen. It has demonstrated efficacy in the treatment of difficult wounds such as diabetic foot ulcers compared with both conservative therapy^44^ and other biological scaffolds containing live cells^45^. One possible explanation for the decreased re-epithelialization observed with the collagen scaffold may be the overall thickness of the injured tissue in our split thickness xenograft model compared with wounded full thickness human skin. Indeed, the thickness of this product was readily observed to create a “bulging” of the epithelial edge in our model and this likely posed mechanical limitations for migration of the epithelium during wound healing. The hydrogels were applied to the wound in such a way to fill it completely from edge to edge, which is a critical step for the desired outcome of improved wound healing, thus the thickness of the swollen hydrogel is identical to the depth of the wound. Otherwise, the presence of gaps between the hydrogel and the wound edge could inhibit epithelial cell migration.

Our previous study was focused on confirming the efficacy of the Q-Peptide hydrogel in a diabetic wound healing model in a rodent, therefore primary cells from a diabetic patient were used for in vitro confirmation of Q-Peptide effects on cell migraiton, with a view of developing therapies for diabetic ulcers^15^. To evaluate if the Q-Peptide hydrogel has a potential to drive re-epithelialization in healthy human skin, we carried out the current study with a view of developing therapies in the future for acute wounds in healthy subjects, such as those resulting from surgeries, donor site grafting or accidents. Therefore, primary normal epidermal human keratinocytes derived from adults were used for these in vitro studies.

Importantly, the accelerated closure of the Q-peptide group was observed in parallel with rapid resolution of keratinocyte proliferation and a return to uninjured thickness of epithelium. This finding is important as the development of epithelial tumors such as squamous cell carcinoma and basal cell carcinoma can develop subsequent to wounding^46,47^, with a hallmark of epidermal tumor promotion being development of hyperplastic epidermis^48^. The rapid return to baseline epidermal thickness observed in the Q-Peptide Hydrogel treated group thus demonstrates that this product was not observed to disrupt the fine balance of wound healing and epidermal overgrowth in this model of wound healing. In contrast, an epidermis that is too thin, as in the non-treated (E) group, can lead to loss of protection^49^. Likewise, the decrease in keratinocyte proliferation observed in the Q-Peptide Hydrogel group may reflect a more advanced stage of healing or the effect of increased migration or survival of epithelial cells rather than proliferation leading to improved re-epithelialization. Taken together, the overall lack of sustained epithelial cell proliferation suggests that the Q-Peptide Hydrogel is unlikely to lead to proliferative disorders of the epithelium, but further safety studies are needed to definitively understand the complete safety profile in humans.

Certainly, animal models of human wound healing are not without limitation. Following full-thickness skin injuries, epithelialization of the wound is essential. Previous ex vivo studies demonstrate that wounding a discarded human skin sample is an established approach to study re-epithelialization upon biomaterial application^50^. The current model is useful in studying human skin re-epithelialization in an in vivo setting^21^, as an important step on the journey from biomaterial development to translation in human use.

Although nude mice have innate immunity, other important aspects such as involvement of the adaptive immune response are absent in this model. Therefore, an autologous large animal model that more closely resembles that of human cutaneous anatomy such as a porcine model will be an appropriate next step in clinical translation. Our previous study in lower limb wounds in the immunocompetent equine model observed no difference in macrophage or total granulocyte recruitment at day 14 in Q-Peptide treated wounds compared to untreated wounds suggesting there was no pathologic dysregulation in immune recruitment. In a further in vitro model, a novel macrophage polarization state consisting of the production of both pro- and anti-inflammatory cytokines was observed when macrophages were cultured on the Q-Peptide Hydrogel^17^. Results from these two previous studies can begin to shed some light on the role of the immune modulation during healing by the Q-Peptide Hydrogel, however future studies should focus on studying the cellular dynamics of the immune system, such as phenotypic composition, time-course, and cytokine/chemokine production during healing.

## Conclusions

Overall, we confirm the Q-Peptide Hydrogel promotes the migration of adult human keratinocytes and modulates their cytokine secretion profile in vitro. Additionally, application of the Q-Peptide Hydrogel during wound healing led to an improved rate of re-epithelialization of human epidermis, a response that is enabled not by increasing proliferation, but rather by transiently increasing migratory capacity and survival of human keratinocytes during the wound healing process. Taken together, these data provide valuable pre-clinical evidence supporting the use of QHREDGS peptide-modified hydrogels for human wound healing applications.

## Materials and methods

### Animals

All animal work received prior approval from the University of Calgary Health Science Animal Care Committee and was in accordance with the Canadian Council on Animal Care Guidelines (approved protocol #AC14-0019). Reporting in the manuscript involving animals follows the recommendations of the ARRIVE guidelines.

### Human split thickness xenograft

Collection and use of human skin samples was approved for use by the Conjoint Health Ethics Review Board at the University of Calgary (approved protocol #REB15-0303) and in accordance with the recommendation outlined in the Declaration of Helsinki. Human skin was harvested and grafted as previously described^22^. Briefly, full thickness adult skin samples were obtained with informed consent from abdominoplasty patients from the Foothills Medical Center in Calgary Alberta. Donor profiles can be found in Table 1. Within 24 h of procurement, the human skin was grafted onto the backs of mice. Full thickness skin wounds (2 cm^2^) were created on the backs of adult athymic (Nu/Nu) mice (n = 50) under isofluorane anesthesia. HSTGs were cut to the exact size and sutured into place using 6–0 polygalactin 910 (Vicryl) in a simple interrupted pattern. Analgesia was provided by intra and postoperative administration of buprenorphine. The wounds were bandaged for 10 days with a foam-based silver dressing (Mepilex, Molnlycke Health Care, Sweden) held securely with and elastic adhesive bandage (Lightplast® Pro, BSN Medical) circumferentially around the abdomen.

**Table 1:** Donor profiles for human split thickness skin grafts.

Donor	Age	Sex
1	35	Female
2	53	Female
3	50	Female
4	40	Female
5	57	Female
6	40	Female

### Wound creation

After 2–3 months of integration, accepted grafts (n = 42) underwent 4 mm full excisional wound creation within the center of the graft, and the wound received one of the following treatments: (1) no treatment, (2) FDA approved collagen scaffold (*Integra Lifesciences*, NJ), (3) Collagen-chitosan gel without the peptide (Peptide-free hydrogel) and (4) Q-Peptide modified gel. N = 4–5 animals per treatment group, per time point, were grafted for a total of 50 initially grafted animals. Some human skin grafts were not successful, but at a minimum N = 3 animals were used per time point, per treatment group for each of the analyses reported in the paper in various figures. A combination of permanent marker around the wound edge and injection of India ink around the wound edge were used to validate the location of the wound edge. The wounds were covered with an adhesive, waterproof dressing (Tegaderm™ transparent film dressing, 3 M™) to ensure a moist wound healing environment and maintain product contact with the wound, but this was found to not adhere well to the nude mice. Thus, an additional circumferential bandage (Lightplast®) was required to ensure maintenance of the test products. This bandage prevented visualization of wounds prior to designated euthanisia. To overcome this, n = 3 mice representing grafts harvested from different human donors were selected for photographing as well as tissue harvest at each timepoint. Wound healing was evaluated by histology and immunohistochemistry at three subsequent timepoints (14, 21, and 28 days post wounding) to allow for temporal observation of wound healing across groups. Wounds were photographed on day 0 immediately after treatment, and on days 14, 21, and 28 with a standard ruler positioned at the level of the wound. Using Fiji (ImageJ, NIH), HSTG contraction was assessed at a day 21 and day 28, to allow for enough time for the effect of treatment to reflect on wound contraction. Specifically, we subtracted wound area on day 0 by day 21, and on day 0 by day 28. These two measurements were performed on different sets on animals, since the euthanization was performed at both day 21 and day 28 for histological analysis. A positive HSTG contraction area signified that the skin grafts had contracted by the day 28 endpoint, and 0 indicated that no skin graft contraction had occurred. A wound closure score was assigned to each healed wound by two blinded observers based on the gross morphological images. On a scale from 0–1, wounds were assigned a score based on the following criteria: open wounds = 0, closed wound with attached eschar (scab) = 0.5, and closed wound with no eschar present = 1.

### Conjugation of peptide to chitosan

Using 1-ethyl-3-(3-dimethylaminopropyl) carbodiimide (EDC; ThermoFisher Scientific Cat#22980) and N-hydroxysuolfosuccinimide (S-NHS; ThermoFisher Scientific Cat#24510) chemistry, the QHREDGS peptide (Q-Peptide; Genscript), or the Scrambled Q-Peptide (DGQESHR; Genscript) was conjugated to chitosan (UP-G213, Novamatrix) as previously described^15,29,51^. Briefly, chitosan was dissolved in 0.9% normal saline, and mixed with a solution of the Q-Peptide (or the Scrambled Peptide), EDC, and S-NHS for 3 h. The reaction solution was diluted, then dialyzed against distilled water for 24 h, and sterile vacuum filtered before lyophilization for 48 h. This recipe has been previously validated to conjugate the Q-Peptide to chitosan at a concentration of 650 μm^29^. The conjugated Q-Peptide-chitosan, and Scrambled-Peptide-chitosan was stored at − 20 °C until use.

### Hydrogel formation

Either the conjugated Q-Peptide-chitosan, or chitosan alone was mixed with type 1 collagen (Corning Cat# CB-40236) at a 1:1 ratio for a final concentration of 2.5 mg/mL. The hydrogel was neutralized to a pH of 7 with 1 M sodium hydroxide, and kept on ice until 10 min before use, where it was brought up to room temperature to initiate gelling. The un-gelled material was applied to the 4 mm wound and gelled in the wound. Upon treatment of the wound, the hydrogel gelled through thermal ionic bonds as a result of the animal’s body temperature.

### Biomaterial characterization

Water-in-air contact angle was measured for the peptide-free hydrogel film and the Q-Peptide Hydrogel film. Briefly, hydrogel films were solvent casted onto glass slides. A droplet of water was deposited onto the films and contact angle (left and right) was measured immediately, and 1 min after droplet formation. Swelling ratio of the hydrogel was measured by pipetting 100 μL of the peptide-free hydrogel and Q-Peptide Hydrogel pre-gel. The hydrogel was crosslinked at 37 °C overnight, flash frozen in liquid nitrogen and lyophilized. Samples were weighed (M_0_). 900 mL of pre-warmed PBS was added, and samples were returned to a 37 °C incubator. At 2 h, 4 h, 6 h, and 24 h, the supernatant was removed, and the hydrogel was patted dry with a Kimwipe, and the samples were weighed. Swelling ratio was calculated as M_t_/M_-_, where M_t_ is the mass at time t, and M_0_ is the starting mass.

### Adult human keratinocyte culture

Primary adult human epidermal keratinocytes (HEKa) were purchased (ThermoFisher Scientific, C-005-5C) and cultures in EpiLife (Gibco Cat#MEPI500CA) medium supplemented with 1% penicillin/Streptomycin (Gibco Cat#15140122) and Epilife Defined Growth Supplement (EDGS; Gibco Cat#S0125). Flasks were coated with a coating matrix (Gibco Cat#R011K) prior to culture and passaged when 70–80% confluence was reached. In all experiments, fourth or fifth passage HEKa were used.

### Adult human dermal fibroblast culture

Human adult fibroblasts (HDF) were purchased from (Angio-Proteomie, Cat # cAP-0008-ad) and cultured using DMEM (Gibco Cat#11960-044) medium supplemented with 1% penicillicin/streptomycin (Gibco Cat # 15140-122) and 10% fetal bovine serum (FBS) (Gibco Cat #12483-020). Cells were cultured to 80–100% confluency before use. In all experiments the cells were used at passage seven through thirteen.

### Solvent casting of hydrogel films

In order to perform the migration assay, the Q-Peptide Hydrogel, Scrambled Peptide hydrogel and the peptide-free hydrogel was solvent casted onto 24 well plates as previously described^15^. Briefly, either the conjugated Q-Peptide-chitosan, Scrambled-Peptide-chitosan or chitosan alone was dissolved in 0.5 N acetic acid, and mixed with collagen at a 1:1 ratio. 24-well plates were coated with 250 μL, and left to evaporate in a biosafety cabinet. Before use, the coated plates were rinsed three times with phosphate buffered saline (PBS).

### Migration assay

Two-chamber culture inserts from ibidiⓇ were carefully placed on the solvent casted films in the 24-well plates. HEKa were seeded at 0.5 × 10^5^ cells/chamber, and allowed to adhere for 2 h. Afterwards the culture inserts were carefully removed and the wells were washed with PBS to rinse away any non-adherent cells. The wells were filled with complete EpiLife medium and supplemented with calcium for a final concentration of 0.12 mM Ca2 + to promote collective migration. The cells were imaged every 2 h for the first 8 h, and the next day at 24 h. The leading epithelial gap was traced using ImageJ, and the area was measured. The gap area was calculated as a percent of original gap area.

### Cytokine analysis by ELISA

Cytokine analysis was performed using ELISA by Eve Technologies (Calgary, AB). The human cytokine array/chemokine array 71-plex panel (sCD40L, EGF, Eotaxin, FGF-2, Flt-3 ligand, Fractalkine, G-CSF, GM-CSF, GROα, IFNα2, IFNγ, IL-1α, IL-1β, IL-1ra, IL-2, IL-3, IL-4, IL-5, IL-6, IL-7, IL-8, IL-9, IL-10, IL-12 (p40), IL-12 (p70), IL-13, IL-15, IL-17A, IL-17E/IL-25, IL-17F, IL-18, IL-22, IL-27, IP-10, MCP-1, MCP-3, M-CSF, MDC (CCL22), MIG, MIP-1α, MIP-1β, PDGF-AA, PDGF-AB/BB, RANTES, TGFα, TNFα, TNFβ, VEGF-A, 6Ckine, BCA-1, CTACK, ENA-78, Eotaxin-2, Eotaxin-3, I-309, IL-16, IL-20, IL-21, IL-23, IL-28A, IL-33, LIF, MCP-2, MCP-4, MIP-1d, SCF, SDF-1A + β, TARC, TPO, TRAIL, TSLP), Human MMP/TIMP array panel (MMP-1 (Collagenase 1), MMP-2 (Gelatinase A), MMP-3 (Stromelysin 1), MMP-7 (Matrilysin), MMP-8 (Collagenase 2), MMP-9 (Gelatinase B), MMP-10 (Matrix Metalloproteinase 10), MMP-12 (Macrophage Metalloelastase), MMP-13 (Collagenase 3), TIMP-1, TIMP-2, TIMP-3, TIMP-4) and the TGF-β 3-plex (TGF-beta 1, 2, and 3). Background media concentration was subtracted from each cytokine concentration.

### LDH assay

Media from HEKa migration was collected at hours 5, 10, and 24. An LDH cytotoxicity assay (CyQUANT LDH Cytotoxicity Assay Kit; ThermoFisher Scientific) was performed as per manufacturer’s instructions. Briefly, collected media was spun down and incubated with the reaction mixture for 30 min. The reaction was stopped with the stop buffer, and the absorbance was recorded using a spectrometer (Cytation 5, BioTek) at 490 nm and 680 nm.

### Immunocytochemistry

Migrating HEKa were fixed at 5 h, 10 h, and 24 h with 4% paraformaldehyde. Human dermal fibroblasts were fixed at 24 h. Cells were stained with the proliferation marker, Ki-67 (Invitrogen Cat# 14-5698-82; 5 µg/mL dilution), the secondary antibody goat anti-rat Alexa Fluor 555 (Invitrogen Cat# A21434; 1/100 dilution) and counterstained with 4′,6-diamidino-2-phenylindole (DAPI; Invitrogen Cat# 1306; 3 µM). The cells were imaged using an Olympus CKX41 inverted microscope and CellSens software (Olympus Corporation). Percentage of proliferating cells was calculated using ImageJ.

### Histology and immunohistochemistry analysis of tissue

Following euthanasia, the wound and surrounding tissue was excised and fixed in 4% paraformaldehyde, OCT embedded, and sectioned into 35 μm thick sections. Sectioning was completed systematically on all wounds such that 4 sections, together representative of a 1 mm span of wound were included on the same slide. This allowed our team to effectively evaluate histologic and immunohistologic characteristics across comparable sections of the wound, including multiple technical replicates (sections) averaged for each analysis and timepoint. The sections were stained with either hematoxylin and eosin (H&E) or Masson’s trichrome by the University Health Network Pathology Research Program laboratory, or immunostained using anti-Ki-67 (eBioscience #14-5698-82;1/200 dilution), anti-human nuclear antigen (hNA; Milipore #MAB1281; 1/200 dilution), anti-Keratin 14 (K14; Covance #PRB-155P; 1/500 dilution), anti-α-smooth muscle actin (α-SMA; Abcam Cat#Ab5964; 1/250 dilution), or anti-CD31 (BD Biosciences Cat#550724; 1/50 dilution), followed by goat anti-rabbit Alexa Fluor 647 (Invitrogen Cat# A21245; 1/500 dilution), or goat anti-rat Alexa Fluor 555 (Invitrogen Cat# A21434; 1:500 dilution). All staining was performed as a “batch” within analyses to ensure effective comparisons are made between groups.

To characterize the quality of healing, H&E stained images were scanned with a ScanScope XT whole slide scanner at 20 × at the Advanced Optical Microscopy Facility (AOMF, Toronto, Canada), and analyzed using Aperio ImageScope (v11, Aperio Technologies). Percent re-epithelialization was calculated by measuring the epithelial gap as the distance between the leading epithelial tongues, and the epithelial thickness was measured at 300 μm from the leading epithelial tongues or at the center of the wound in re-epithelialized tissue. Rete ridge ratio was measured as the length of the epidermis:dermis boundary/length of the top of the epidermis. Rete ridge ratio of 1 indicates no rete pegs, and a rete ridge ratio greater than 1 indicates the reformation of rete pegs.

H&E stained tissues were scored by blinded observers using a previously published system^24^ to characterize features of wound healing, such as quality of granulation tissue, presence of inflammation, neovascularization and re-epithelialization. In this system the scores range from 1 (no healing) to 12 (complete healing)^24^, Ki-67 stained tissue sections were imaged using a Zeiss Observer microscope and the percentage of positively stained Ki-67 cells were quantified by a blinded observer then calculated as a percentage of total cells using a DAPI counterstain in the epithelial tongue.

CD-31 and α-SMA stained slides were imaged using Nikon A1R Resonance Scanning Confocal and Nikon NIS Elements C Software. Vessels/area and vessel diameter wasmeasured using ImageJ^52^. Mature and stable blood vessels were identified not only by their distinct morphology, but also by co-expression of both CD31 and α-SMA. Quantification and measurement of diameter was performed by a blinded observer and completed for both the center of the wounded area as well as the surrounding, uninjured area for each graft. Vascularity measures were then expressed as the percent of each wound’s own uninjured graft tissue to eliminate any variability across individual grafts.

Additionally, a scoring system based on intensity of staining was then used to qualitatively explore the α-SMA staining not associated with discrete vasculature (0 = no staining, 0.5 = slight, 1 = moderate, 2 = intense staining).

### Statistical analysis

All results are presented as mean ± SD. Statistical analysis was performed using GraphPad Prism 6. Normality and equality of variances was tested using Shapiro–Wilk test and Brown-Forsythe test respectively. Significance was calculated using either a one-way ANOVA, or a two-way ANOVA where appropriate. To identify significant differences between experimental groups, a Tukey’s multiple comparisons test was employed. A value of *p* < 0.05 was considered statistically significant. N = 3–5.

## Supplementary Information

Supplementary Information.

## Data Availability

The datasets used throughout this work are available from the corresponding author(s) upon reasonable request.
