# Supplementary material for: Application of an instructive hydrogel accelerates re-epithelialization of xenografted human skin wounds
Source: Sci Rep. 2022 Aug 20;12:14233. doi: 10.1038/s41598-022-18204-w (PMC9392759; doi:10.1038/s41598-022-18204-w)
Supplement: Supplementary file 1 — Supplementary Information. [file 41598_2022_18204_MOESM1_ESM.pdf]

Supplementary Materials

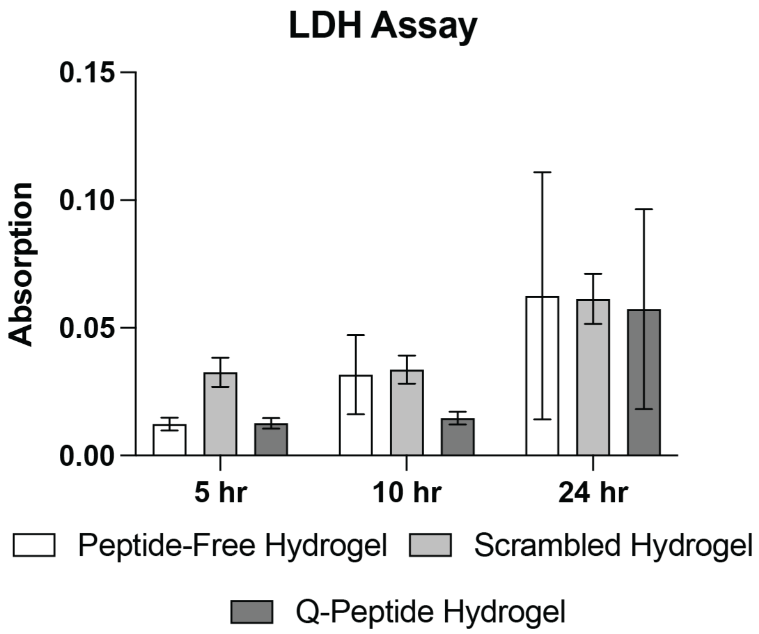

Supplemental Figure 1: LDH Assay confirms no significant differences between groups on HEKa cells on the peptide-free hydrogel, Scrambled Peptide Hydrogel, and the Q-Peptide Hydrogel. n=3-5.

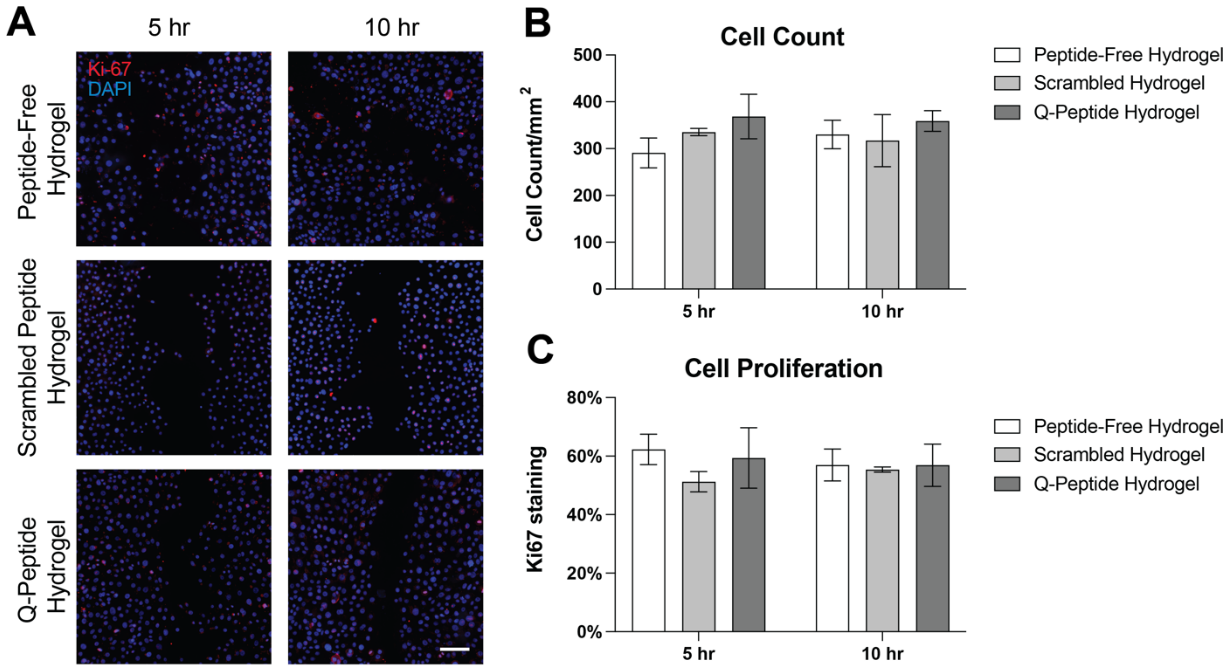

Supplemental Figure 2: Ki-67 staining (red) with DAPI counterstain (blue) for A) migrating HEK cells at 5 and 10 hours cultured on the Peptide-Free Hydrogel, Scrambled Peptide Hydrogel, and the Q-Peptide Hydrogel. SB = 200μm. B) Quantified DAPI stained cells per area at 5 hour and 10 hour shows no difference between the three culture conditions. C) Quantification of positively stained Ki-67 HEKa cells at 5 and 10 hours. N = 3. Data are presented as mean ± SD.

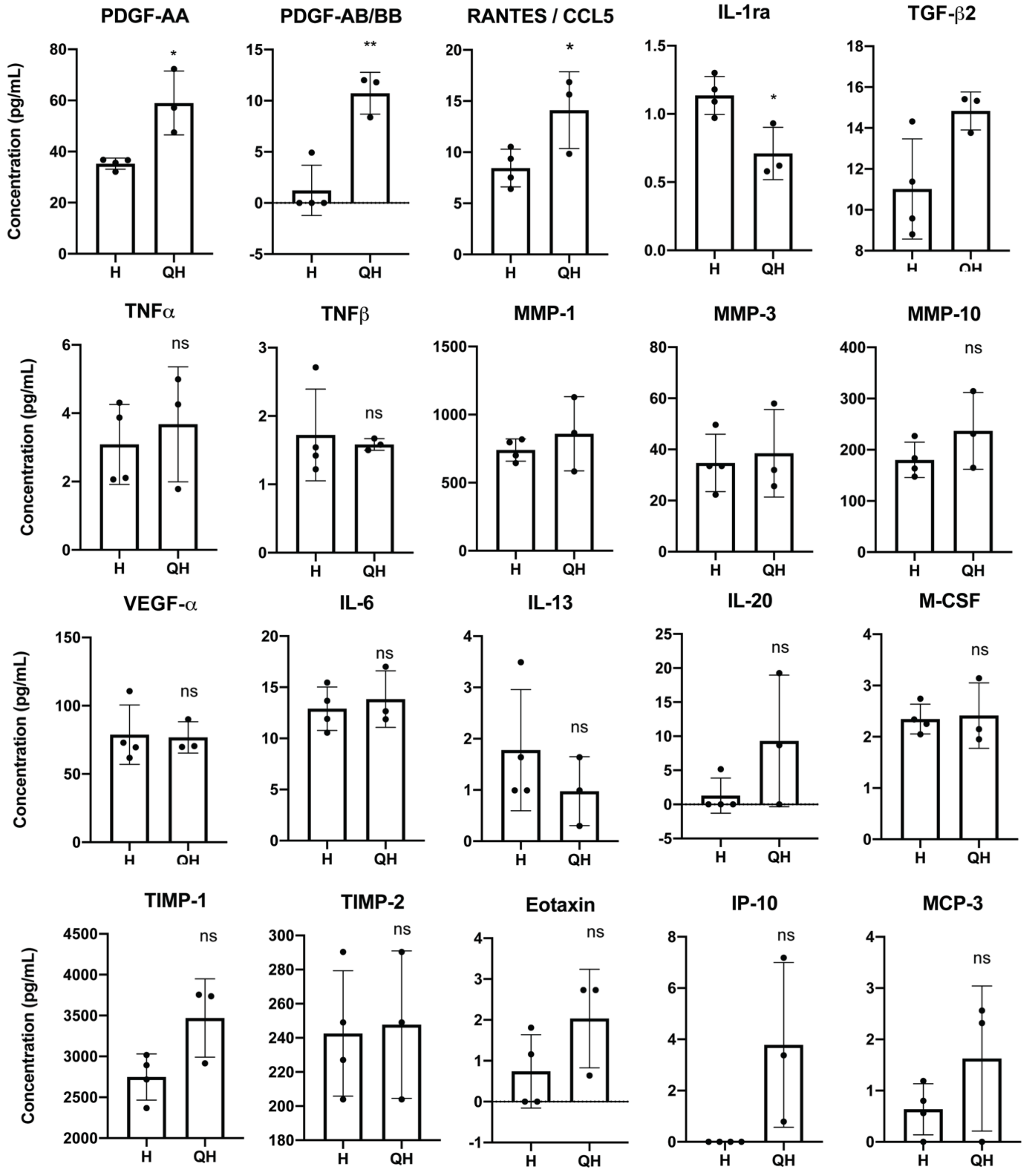

Supplementary Figure 3: Concentration of proteins in media after aHEK culture in the presence or absence of Q-Peptide Hydrogel (continued from figure 1). Proteins found to be present at or above 1pg/mL in at least one treatment group are included. Background media control is subtracted from sample concentrations. Data are presented as mean ± SD. * = $p < 0.05$, ** = $p < 0.01$. H = Peptide free Hydrogel, QH = Q-Peptide Hydrogel, N=3

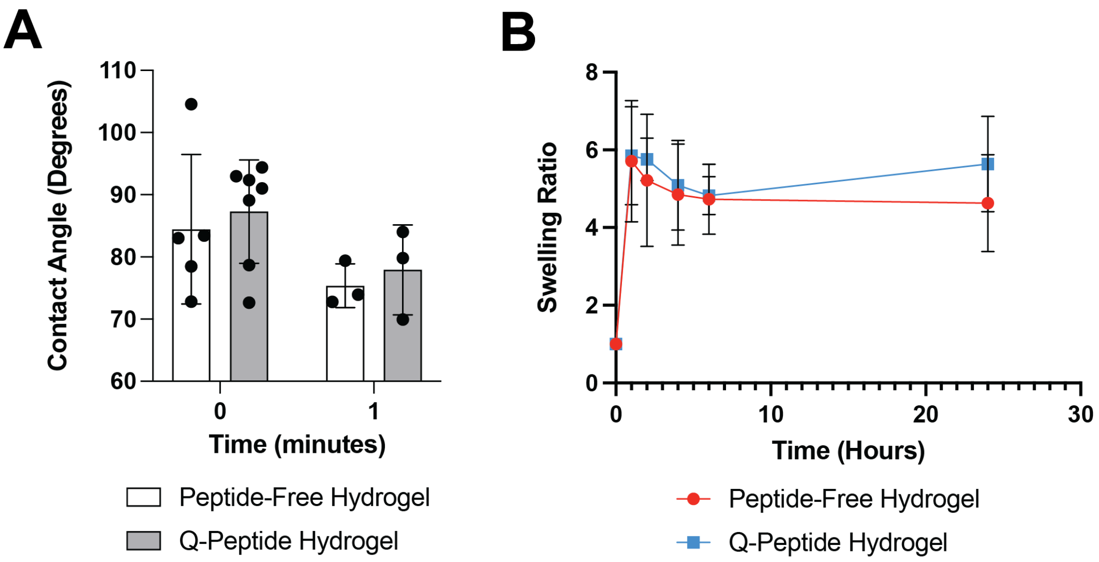

Supplementary Figure 4: Hydrogel hydrophilicity characterization. A) Contact angle was measured on the peptide-free hydrogel film (white) and the Q-Peptide Hydrogel film (grey). Contact angle was measured immediately after droplet formation, and 1 minute after droplet formation. n = 3-7. B) Swelling of the hydrogel was measured over 24 hours for the Peptide-free hydrogel (red) and the Q-Peptide Hydrogel (blue). Following gelation and lyophilization, hydrogel reaches steady state within 24 hours. n = 5. Data are presented as mean ± SD.

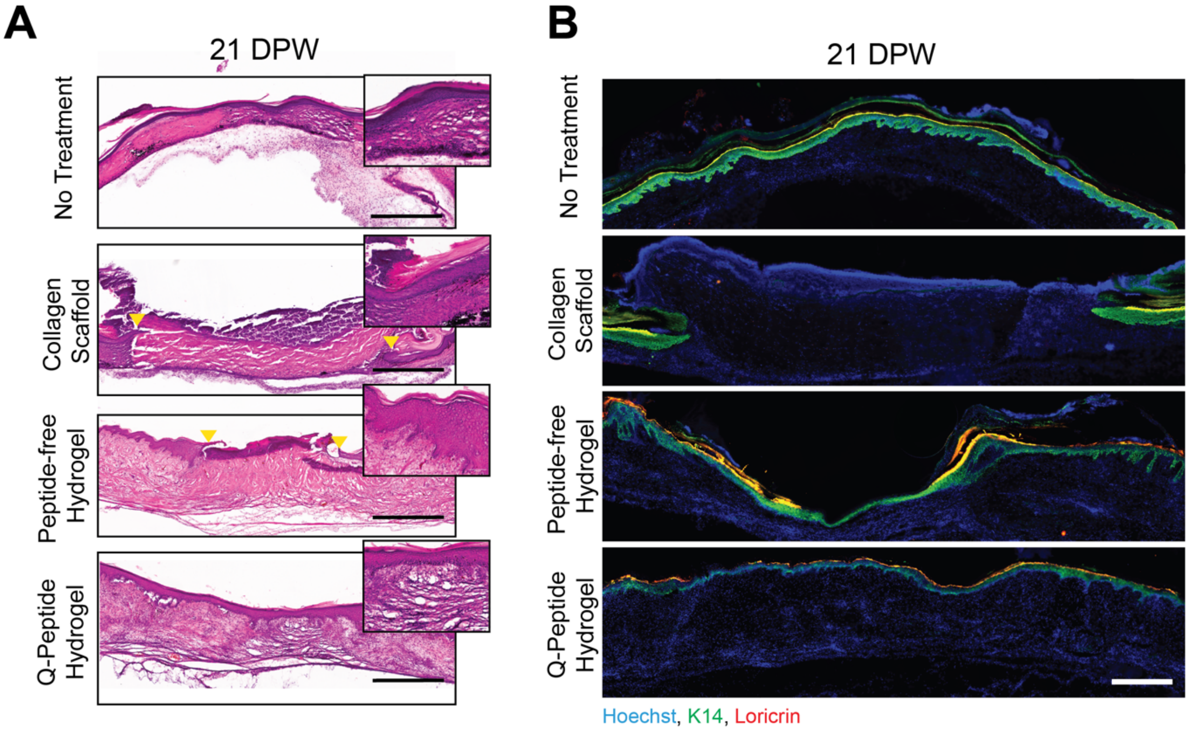

Supplementary Figure 5: Day 21 wound histology and immunostaining. (A) Representative Hemotoxylin and Eosin stained cryosections from the middle of wounds at 21 days post wounding (DPW). Yellow arrow heads indicate measurements used for wound gap. SB = 1mm. Inset SB = 0.1mm B) Immunostained tissue sections on day 21 for K14 (Green), loricin (Red) and Hoechst (Blue). SB = 500μm.

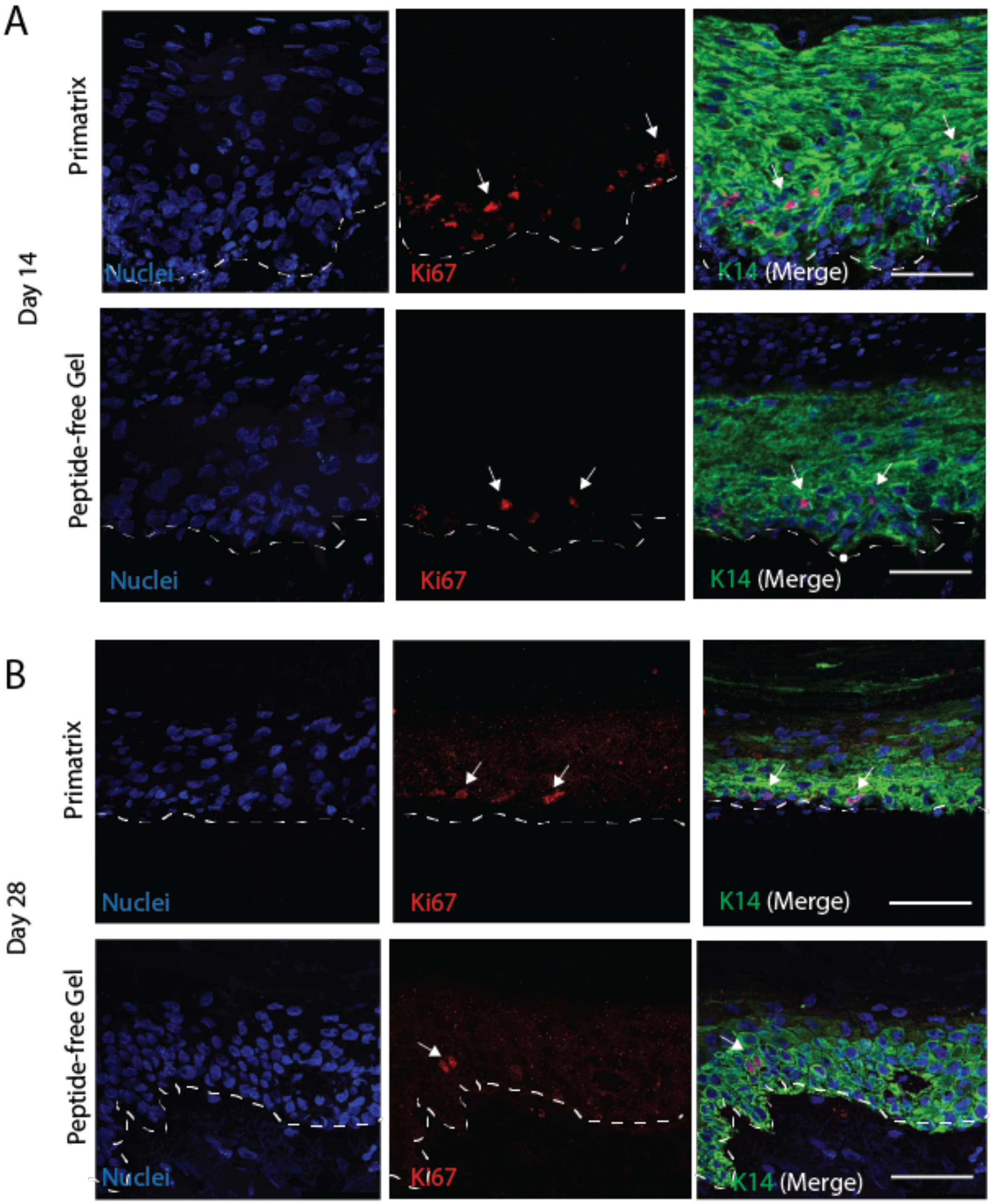

Supplementary Figure 6: Immunohistochemistry of Ki67 at A) d14 and B d28) in groups not shown in figure 2. Dashed line indicates basal epithelial layer. Arrows show Ki67 positive nuclei. Scale bar 100 µm

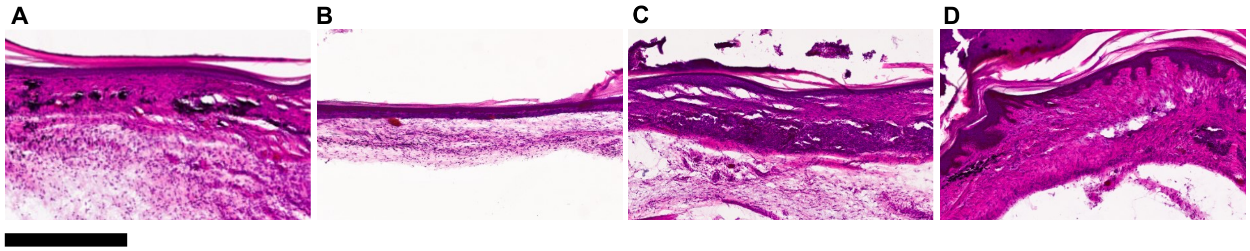

Supplementary Figure 7. High magnification images of H & E staining illustrate the formation of rete ridges in the Q-Peptide Hydrogel treated human split thickness graft 28 days after wounding. A) No treatment B) Collagen Scaffold C) Peptide-free Hydrogel D) Q-Peptide Hydrogel. Scale bar 700mm.

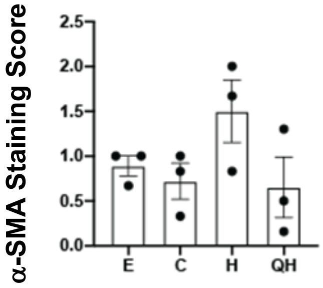

Supplementary Figure 8. Scoring for a-SMA presence in different treatment groups. E no treatment, C Primatrix control, H Peptide-free hydrogel and QH Q-peptide hydrogel.

Sample	Mean (%)	SD	N	T-Test
				Control
Control	53.96	7.82	3	
Q-Peptide Hydrogel	42.24	2.52	3	0.0687

Supplemental Table 1: Quantification of positive Ki-67 staining (%) revealed no significance between Q-Peptide Hydrogel (QH) and tissue culture plastic control after 24 hours with human dermal fibroblasts. Statistical Analysis included t-test.
